# The impact of place attachment on the environmentally responsible behavior of residents in National Park gateway communities and the mediating effect of environmental commitment: a case of China National Park

**DOI:** 10.3389/fpsyg.2024.1386337

**Published:** 2024-08-26

**Authors:** Xiangxiang Xie, Zenong Wang

**Affiliations:** College of International Tourism and Public Administration, Hainan University, Haikou, China

**Keywords:** place attachment, environmentally responsible behavior, environmental commitment, national park, gateway community

## Abstract

As a member of the national park, the environmentally responsible behavior of the entrance community residents has an important impact on the ecological protection of the national park. However, there are still insufficient studies on the mechanism of influence of community residents’ place attachment on environmentally responsible behavior and the role of environmental commitment. Based on the theory of interdependence, this study explored the impact of residents’ place attachment in a national park gateway community on environmentally responsible behavior, and examined the mediating role of environmental commitment. We conducted empirical research on Shuiman gateway community of the Hainan Tropical Rainforest China National Park. Through structural equation modeling, the survey of 375 residents yielded the following conclusions: (1) place dependence indirectly affect environmentally responsible behavior via the mediating effect of place identity; (2) both place dependence and place identity have a positive and significant impact on environmental commitment; (3) environmental commitment has a positive and significant impact on environmentally responsible behavior; (4) the mediating effect of environmental commitment on the impact of place dependence on environmentally responsible behavior is significant and “place identity–environmental commitment” also has a significant mediating effect. Finally, three managerial insights were discussed. This study enriches the connotation and application scope of interdependence theory, explains the different roles of place dependence, place identity, and environmental commitment in the study of environmentally responsible behavior, and provides some inspiration for future research and practice of environmentally responsible behavior. It is helpful for community residents to participate in the ecological environment governance of national parks and achieve sustainable development.

## 1 Introduction

The harmonious coexistence of man and nature has become the direction for China and a necessity for the construction of China’s national park system, and it encapsulates the maintenance of the originality and integrity of national parks (Zhao and Yang, 2021). Therefore, implementation of the “ecological protection first” concept in national parks requires the cooperation and mutual benefit of all stakeholders in the national park community, including community residents. Exploration of the factors and mechanisms which influence the environmentally responsible behavior of stakeholders in national parks has an important and far-reaching impact on the sustainable and healthy development of national parks and is of great practical significance in the achievement of the goal of “carbon neutrality and carbon peaking” globally.

Some villages and towns close to national parks are oriented to meet the needs of the national parks and have grown alongside the development of national parks, gradually evolving into national park gateway communities. Current research on the behavior of residents in national park communities tends to generalize the communities surrounding national parks, ignoring the differences between national park gateway communities and internal communities (Chen et al., 2021). Such research also ignores the differences between the neighboring villages and towns, which are dominated by traditional agriculture, and the gateway communities, which are vigorously developing green agriculture and ecotourism (Bai et al., 2023). Location differences across townships can significantly affect the perception of members of the community (Zhou et al., 2017). The particular location and policy of the gateway community give it a special functional positioning which may lead to the emergence of types of environmentally responsible behaviors among residents which differ from those of other communities. It is necessary to differentiate and study these individually.

Research on environmentally responsible behavior has gradually matured in recent years as it has attracted more attention, shifting from explaining individual behavior in terms of a single rational–cognitive factor to a multifaceted perspective in which rational and emotional factors work together (Niu and Liu, 2022). However, in current research on the factors influencing environmentally responsible behavior, exploration of emotional factors is still thin compared with that of diversified rational cognitive factors. Some studies have shown that emotional factors tend to have a stronger driving effect on individual behavior than rational cognitive factors (Wang and Wu, 2015). Besides, a greater number of studies have investigated the environmentally responsible behavior of tourists than that of residents. It is therefore necessary to pay more attention to community residents.

Place attachment is a classic theory that elaborates on human–place relations. This theory provides a feasible path for research on environmentally responsible behavior. Most current studies use it as a mediating variable to explore the direct influence on environmentally responsible behavior, and there is a lack of in-depth discussion on the specific mechanism of place attachment and environmentally responsible behavior (Huo et al., 2023). According to the theory of interdependence, the dependence of an individual on a certain place will lead to a greater commitment to the place, thus stimulating the emergence of relevant behaviors (Rusbult, 1980). This theory remains valid in the context of people and the environment and has given rise to the concept of environmental commitment (Davis et al., 2008). The human–environment interdependence emphasized in environmental commitment makes it possible to use environmental commitment as a mediating variable to explain the effect of place attachment on environmentally responsible behavior and to take a new approach to the study of factors influencing environmentally responsible behavior.

Based on the above analysis and in order to provide a theoretical basis for the sustainable and coordinated development of the national park and the gateway community, this study took the residents of the gateway community of the national park as its research object, explored the mechanism of place attachment mediated by environmental commitment on environmentally responsible behavior, and carried out empirical tests involving the gateway community of the Hainan Tropical Rainforest China National Park as an example.

The main contributions of this study are as follows. Firstly, this study introduces the interdependence theory and environmental commitment, which are not widely concerned by scholars, into the impact mechanism of environmentally responsible behavior, builds a model of the impact mechanism of environmentally responsible behavior, and takes residents in gateway communities of national parks as the research object to broaden the application scope of environmental commitment. Secondly, this study found the different roles of residents’ place dependence and place identity on environmentally responsible behavior in the context of gateway communities, and analyzed the causes of this relationship according to the location and functional characteristics of gateway communities and local cultural factors, which enriched the research on the differences between place dependence and place identity. Thirdly, through the discussion of two emotional factors, place attachment and environmental commitment, this study emphasizes the important role of emotional factors in the study of environmentally responsible behavior, and has a certain guiding effect for future related research. According to the research conclusion, this study puts forward suggestions for entrance community managers from three aspects: industrial revitalization, cultural cultivation and environmental awareness, which is conducive to promoting the sustainable and coordinated development of national parks and community residents.

## 2 Literature review

### 2.1 National Park gateway communities

China National Parks are state-led, ecological conservation-first, nationally representative ecological regions, and their rich conservation, scientific research, education, recreation, and community development functions have been recognized by scholars (Liu, 2017). Numerous studies involving national park communities, but there is a lack of attention given to the gateway communities of national parks (Wu and Wang, 2022), which are generally regarded as belonging to the same category as the communities inside national parks. Zhu et al. (2021) proposed that the development of communities with different characteristics, including the internal communities and gateway communities of national parks, should be guided by implementation of different categories and policies. Cai et al. (2020) suggested that a four-level development model should be established, including communities within national parks and gateway communities, but discussion regarding the different ecological protection responsibilities of each level was lacking. Wu and Wang (2022) argued that the development of gateway communities in the protected areas of China was not aligned with international standards and needed to be supported by land policies.

Table 1 shows the differences between the different types of communities within and outside national parks. Gateway communities are usually located on the periphery of national parks and are the first stop for tourists coming to national parks, serving as a link connecting national parks, community residents, and tourists. However, internal communities are located within national parks or in the core protected areas and therefore have a weaker connection with external society. The Interim Measures for the Management of China National Parks strictly control human activities within national parks. On the other hand, the Overall Program for Establishing the China National Park System endorses and fosters the expansion and creation of gateway communities. This leads to a more diverse industrial system within the gateway community, which performs not only an ecological protection function but also offers a range of other services such as exhibitions, popular science education, tourism distribution, industrial concentration, and population migration. Consequently, it generates greater social and economic benefits while simultaneously having a less detrimental impact on the ecological environment of the national parks. Compared with the gateway communities, the neighboring ordinary communities which are farther away from the national park are less affected by the national park, and their industrial systems and functional positioning develop according to their situation.

**Table 1:** Differences in different types of communities within and outside National parks.

	Internal communities of national parks	National Park gateway communities	Neighboring ordinary communities
Geographical position	Core protection area or general control area of national parks	Periphery of national parks	Farther away from national parks relatively
Linkages with the outside world	Weak	Strong	Weak or strong (It depends on its geographical location, industry type, culture and other factors)
Degree of constraint by relevant laws and regulations of national parks	Strong	Weak	Weaker
Industrial system	Necessary and limited productive and life activities	Service-oriented, production-oriented, R&D-oriented and other diversified characteristic eco-industries	Traditional/modern agriculture predominates
Functional positioning	Ecological conservation, scientific research, etc.	Ecological protection, cultural exhibition, popularization of science and education, scientific research services, tourism distribution, industrial agglomeration, population relocation, etc.	Building a livable, workable and beautiful countryside according to local conditions

The different development patterns of different types of communities also lead to differences in the behavior of residents (Zhou et al., 2017). Current research has paid less attention to the behavior of residents in gateway communities and their role in ecological conservation. The unique position and value of national park gateway communities are likely to influence the awareness and behavior of residents, which in turn affect the emergence of environmentally responsible behaviors.

### 2.2 Environmentally responsible behavior

The connotations of environmentally responsible behavior can be summarized as the reduction of environmental damage and enhancement of sustainability (Sivek and Hungerford, 1990). Theories such as the theory of planned behavior, norm activation theory, and their extension theories are widely used (Wang and Wu, 2015). At present, the influencing factors of environmentally responsible behavior can be primarily divided into objective contextual factors and subjective individual factors (Chiu et al., 2014). The subjective individual factors include rational cognitive factors and emotional factors (Niu and Liu, 2022), such as service quality and satisfaction (He et al., 2021), place attachment (Jia and Lin, 2015), fear of the environment (Niu and Liu, 2022), and environmental commitment (Wang et al., 2023).

From the point of view of the research object, the current research has focused on the environmentally responsible behavior of tourists and paid less attention to the residents of tourism locations, neglecting the key role of community residents in environmental protection. As the “masters,” community residents in tourism locations are inextricably linked with the local ecological environment. Residents plays an important role in the ecological protection of the community and the surrounding environment (Su and Swanson, 2017). The role and function of gateway community residents in the construction of the national park system in China, which is still at an early stage, require further exploration. In addition, because of the relatively late start of the study, the current exploration of emotional factors remains inadequate: on the one hand, the exploration of potential emotional factors is not sufficiently extensive, and, on the other, the research on the specific mechanism of emotional factors on environmentally responsible behavior is not sufficiently thorough (Wang and Wu, 2015).

### 2.3 Place attachment

Place attachment refers to the emotional connection between people and their place of residence and is a positive human–place relationship (Williams and Roggenbuck, 1989; Qi et al., 2018). Scholars have since expanded the application of place attachment to the relationship between an individual and a particular place (Gibbons and Ruddell, 1995). It is widely recognized that place attachment is a multidimensional concept (Cheng and Wu, 2015), which Williams and Roggenbuck (1989) divides into two dimensions: place dependence and place identity. Place dependence focuses on the fulfillment of material needs in a real sense and represents the functional attachment of the individual to the place (Xi et al., 2021). Place identity represents the emotional love that people have for a place, similar to the love that people have for others, which embodies acceptance of the symbolic and abstract qualities of the place (Pan et al., 2014).

Research has found that place attachment has a multifaceted impact on the behavioral decisions of individuals. Qian et al. (2010) found that place attachment had a positive effect on the shopping behavior of tourists. The effect of place attachment on environmentally responsible behavior has also been widely studied, with both direct and indirect effects (Jia and Lin, 2015; Zhou et al., 2014). At present, there is no academic consensus on the stronger influence of place attachment and place identity on environmentally responsible behavior. A study by Fan et al. (2014) on tourist resorts concluded that place dependence had a greater impact on the environmentally responsible behavior of tourists, whereas the study by Qu et al. (2020) on mass tourism in mature tourist destinations concluded that place identity had a stronger influence on the proenvironmental behaviors of tourists.

### 2.4 Environmental commitment

Environmental commitment is a commitment made by an individual to protect the environment, taking into account his or her relationship with the environment (Wang et al., 2023). The concept is derived from the interdependence theory and Rusbult’s commitment model (Rusbult, 1980, Rusbult, 1983). Dependence refers to the extent to which an individual relies on a relationship partner to satisfy important needs, and commitment is a subjective experience of dependence that directly affects individual behavior. The behavior of committed individuals in a relational setting is influenced by the degree of closeness with the relational partner. Greater dependence will lead to greater commitment to the relationship partner (Rusbult, 1980). Just as two people may influence the well-being of each other, humans and the natural environment have an interdependent relationship (Davis et al., 2008). Davis et al. (2008) extends the applicability of this theory and model to the human–environment relationship, inferring that the greater the level of intimacy an individual has with the environment, the greater their willingness to make environmental commitments. Environmental commitment is the emotional attachment of an individual to their surrounding environmental atmosphere and is a psychological attachment to the long-term orientation of the environment (Davis et al., 2008; Davis et al., 2011). Existing studies have proved that environmental commitment has a significant effect on corporate environmentally responsible behavior (Davis et al., 2011), green product innovation performance (Xing et al., 2020), and levels of environmental management (Wei et al., 2013).

There have been few studies on environmental commitment, and most of them focus on the environmental commitment made by enterprises and their employees, whereas the environmental commitment of residents in the gateway communities of national parks and their influencing factors have not been sufficiently explored. The forces binding gateway communities are not as strong as those in enterprises, and their environmental commitments may therefore differ from those of enterprises.

## 3 Research hypotheses

### 3.1 Impact of place attachment on environmentally responsible behavior

The influence of place attachment on environmentally responsible behavior is supported by numerous studies. These studies confirm that place attachment is a potentially important factor driving environmentally responsible behavior in specific contexts (Gosling & Williams, 2010; Hernandez et al., 2010). When individuals develop a positive attachment to place, environmentally responsible behaviors are more likely to arise (Burley et al., 2007; Walker & Ryan, 2008). Using tourists as research subjects, Jia and Lin (2015) and Wan et al. (2014) confirmed the effects of place attachment on environmentally responsible behavior and its tendency, respectively. The driving effect of place attachment on environmentally responsible behavior is not only reflected in the context of tourism places such as wetland (Lee, 2011), island (Qu et al., 2019) and national parks (Ramkissoon et al., 2013), but also plays a positive role in the context of community residents (Zhang et al., 2014). Taking community residents as research subjects, Liu et al. (2017) confirmed that place attachment and place identity of oasis city residents had a direct effect on compliance and proactive environmentally responsible behaviors, respectively. In the scenario of a national park gateway community, residents born and raised there develop a functional attachment to their place of residence and form an affective identity in their long-term interaction with the place. This attachment prompts residents to develop a sense of responsibility for the place, such as concern for and protection of the environment. Based on this, our study follows the common practice of dividing place attachment into two dimensions, place dependence and place identity, and proposes the following hypotheses:

*H1a*: The place dependence of national park gateway community residents positively influences their environmentally responsible behavior.

*H1b*: The place identity of national park gateway community residents positively influences their environmentally responsible behavior.

### 3.2 Impact of place dependence on place identity

Many scholars have argued that the dimensions of place dependence and place identity are not juxtaposed but sequential, with place dependence tending to emerge earlier than place identity. Pan et al. (2014) pointed out that tourist satisfaction drove the formation of place dependence, whereas place identity took a long time to crystallize from human–place interaction. Lu et al. (2014) demonstrated that place dependence had a significant effect on place identity in urban residents. Uesugi & Kudo (2020) and Kuo et al. (2021) verified that tourists’ place dependence has a significant impact on place identity. As residents of national park gateway communities have been living near the national park for a long time, with the passage of time and frequency of contact, they have developed functional place dependence and emotional place identity. Although the two coexist in community residents, according to previous research and practical experience, place dependence is likely to have an impact on place identity. At the same time, based on the ideas and hypotheses above, place dependence may also have an impact on environmentally responsible behavior through the mediating role of place identity. The studies of Moore & Graefe (1994) and Vaske and Korin (2001) show that place dependence affects people’s attitude towards environment and daily behavior mainly through the mediating role of place identity. Halpenny (2010) found that tourists’ place dependence on national parks can effectively promote place identity, and place identity plays a significant mediating effect on the influence of place dependence on pro-environmental behavior intention. Tang et al. (2008) and Fan et al. (2014) came to a similar conclusion that under the mediating role of place identity, place dependence has a more significant effect on the pro-environmental behaviors of residents and tourists. The following hypotheses are therefore made:

*H2*: The place dependence positively influences the place identity among residents of national park gateway communities.

*H3*: The place identity of residents in national park gateway communities mediates the role of place dependence in influencing environmentally responsible behavior.

### 3.3 Impact of place attachment on environmental commitment

According to Davis et al. (2008), environmental commitment is the psychological attachment and long-term orientation of an individual toward the natural world. Successful long-term oriented relationships eventually lead to higher commitment (Morgan & Hunt, 1994). This definition reflects that the source of environmental commitment is the individual’s place attachment, which goes along with the idea of place attachment theory (Junot et al., 2018). Tournoist’s study of people living in post-socialist cities shows that place attachment and place identification are closely related to commitment (2020). Places can foster a sense of commitment and responsibility (Zhang et al., 2014). In terms of environmental protection, place attachment strengthens the connection of the individual with nature, which leads to environmental commitment. Therefore, this study puts forward the following hypotheses:

*H4a*: The place dependence of national park gateway community residents positively influences their environmental commitment.

*H4b*: The place identity of national park gateway community residents positively influences their environmental commitment.

### 3.4 Impact of environmental commitment on environmentally responsible behavior

Commitments are thought to yield substantial and durable behavior change (Cialdini, 2009). Commitment is a credit guarantee for behavior, and people always tend to honor their commitments rather than violate them. Environmental commitment is an effective factor in stimulating environmentally responsible behavior, and a high level of environmental commitment will prompt individuals to make efforts to protect the environment, even to the detriment of their own interests (Davis et al., 2008). Studies have found that the environmental commitment made by various groups can affect the generation of environmentally responsible behaviors. Afsar and Umrani (2020) found that Employee environmental commitment has a positive association with pro-environmental behavior. Some scholars have demonstrated that the use of commitment tools can motivate hotel guests to engage in environmental behavior (Terrier & Marfaing, 2015; Baca-Motes et al., 2013). A study by He et al. (2022) found that the environmental commitment of tourists positively affected their environmentally responsible behavior. It can be hypothesized that environmental commitment made by residents of national park gateway communities will also be translated into practical actions to protect the environment and protect their credibility. Therefore, the following hypothesis is proposed:

*H5*: The environmental commitment of national park gateway community residents positively influences their environmentally responsible behavior.

### 3.5 The mediating role of environmental commitment

According to interdependence theory, the dependence of an individual on a place (this study used place attachment as an evaluation index of dependence) leads to a greater commitment to the place, and this greater environmental commitment can affect environmentally responsible behavior. The existing literature has proved that environmental commitment plays a mediating role between individual perception and behavior (Coy et al., 2013; Su et al., 2019). Lee (2011) found that environmental commitment mediated the effects of place attachment and recreational participation in environmentally responsible behavior through a survey of wetland tourists in Taiwan. Tonge et al. (2014) argued that the effect of place attachment on environmentally responsible behavior increased with the level of commitment. However, neither of these two studies considered the different roles that place dependence and place identity may play in influencing environmental commitment and environmentally responsible behavior. To maintain and strengthen the interdependent links between individuals and places, residents make a series of commitments in favor of local development, including a commitment to environmental protection, out of functional dependence on and emotional identification with the national park gateway community. This environmental commitment, in turn, is transformed from ideological beliefs into practical actions by residents, promoting the emergence of environmentally responsible behaviors. Therefore, the following hypotheses are proposed:

*H6*: The environmental commitment of national park gateway community residents positively mediates the effect of place dependence on environmentally responsible behavior.

*H7*: "Place identity–environmental commitment" positively mediates the effect of place dependence on environmentally responsible behavior.

**Figure 1:**
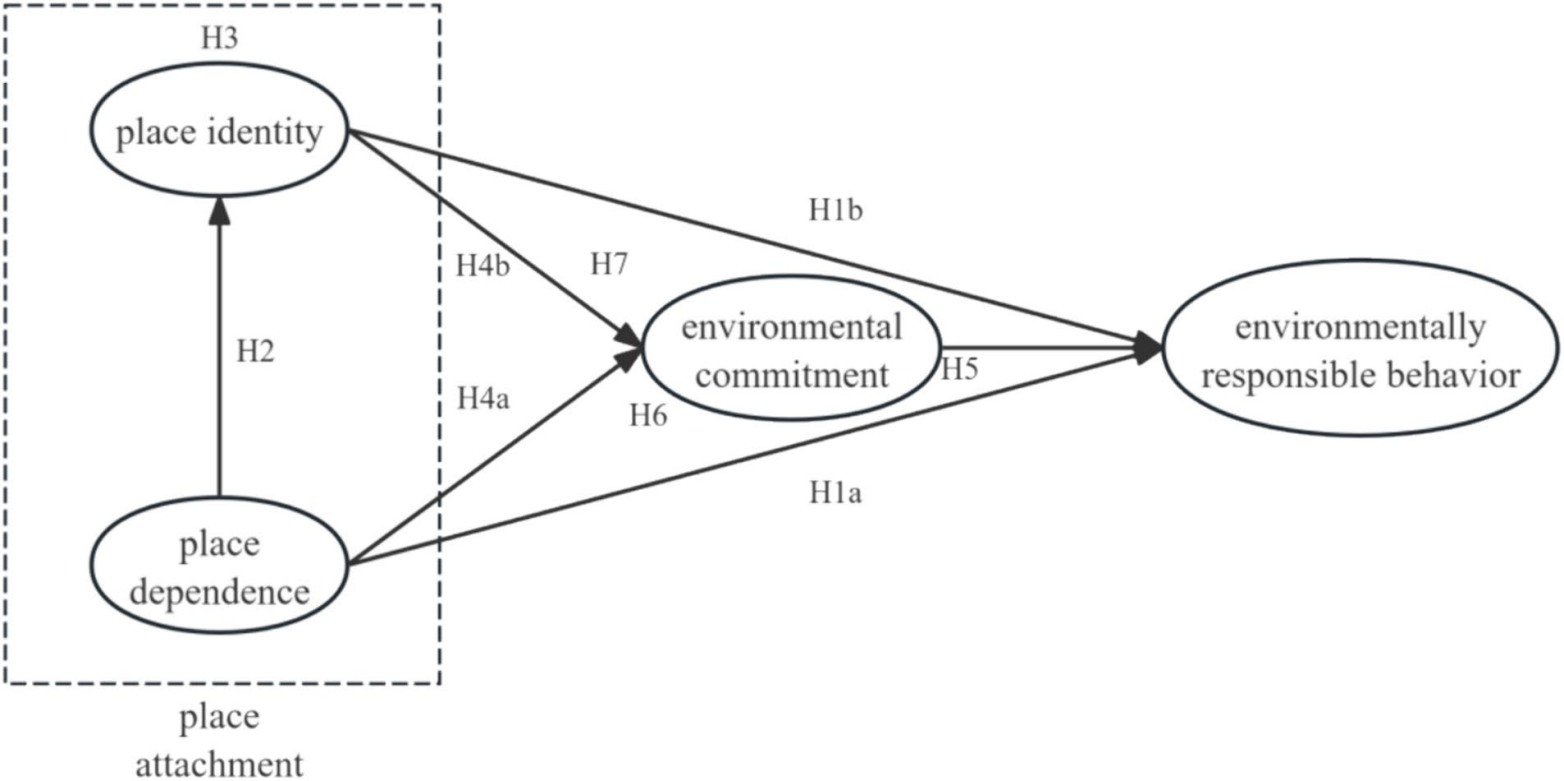
Conceptual model.

Figure 1 shows the conceptual model framework.

## 4 Methodology

### 4.1 Brief description of the study area

**Figure 2:**
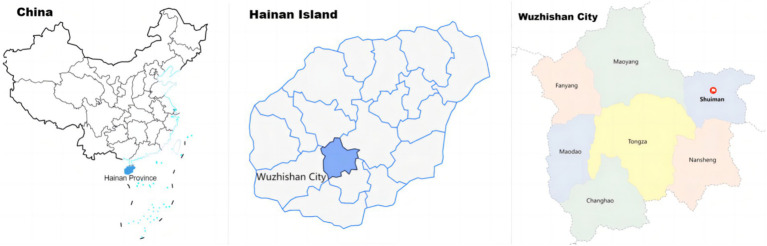
Maps of study area.

**Figure 3:**
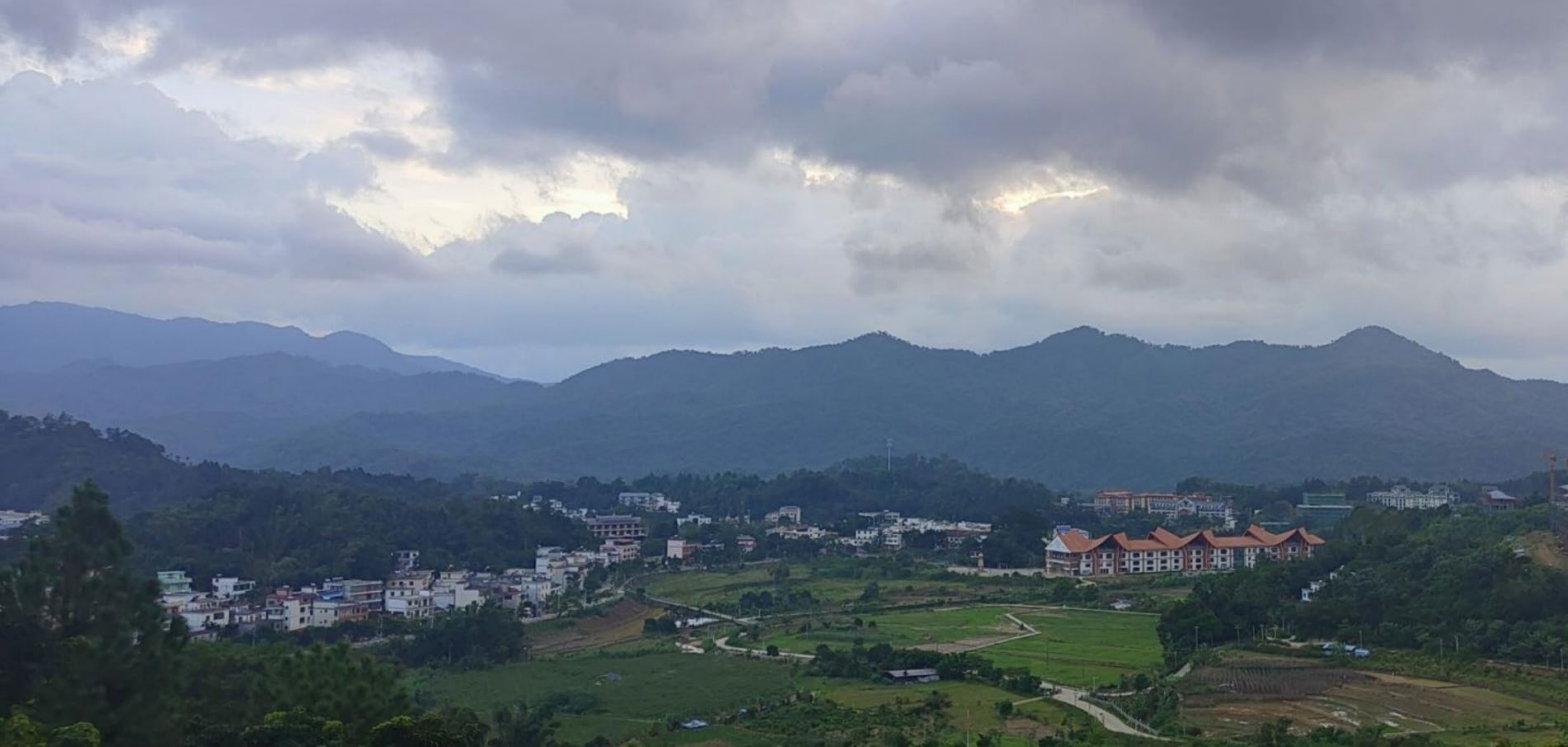
Photo of study area.

Shuiman Township (see Figures 2, 3), Wuzhishan City, Hainan Province, China is the main gateway community of the Wuzhishan Area of the Hainan Tropical Rainforest China National Park. Since July 2023, the Hainan Provincial government and the Wuzhishan City government have opened the bidding work for the Shuiman gateway community project, and the construction of the Shuiman gateway community has made substantial progress. Shuiman Township is located at the foot of Wuzhishan Mountain and surrounded by mountains. It is the highest township in Hainan Island, located in the northeast of Wuzhishan City and 34 kilometers away from Wuzhishan City. The permanent population is 3,992, of which 97.82% are Li and Miao minorities. The Shuiman gateway community occupies is a territory of tropical primitive rainforest with an excellent climate and rich tourism resources, and the forest coverage rate reached 89.13%. The meaning of “Shuiman” in Li language is “very ancient and supreme,” indicating that most of the permanent residents of Shuiman Township are Li and Miao minorities who have been living there for generations. Wuzhishan Mountain and the surrounding tropical rainforest have become emblematic and spiritual symbols of Li and Miao people. They have always maintained a respect for nature while utilizing it for their subsistence. In recent years, the awareness of ecological protection of the residents has been improved through publicity to households and the appointment of forest rangers. There are 5 villages in Shuiman Township, among which Maona Village is a typical representative of the ecological environment and community development of the gateway community of Shuiman Township. The Chinese President’s visit also proved that Maona Village has made outstanding achievements in ecological protection and community development. Maona Village has promoted the sustainable development of the community through the integration of tea-based agriculture and tourism. The owner of the gateway community of the national park has become an important identity generally recognized by residents. After our preliminary research, we found that the vast majority of community residents believe that their community and the National Park are inseparable, and residents have to consider the impact of the National Park on them when living, production and ecological protection in the community. Therefore, Shuiman Township is a suitable representative site for this research.

### 4.2 Questionnaire design

Questionnaire items and sources are given in Table 2. In this paper, the place attachment scale designed by Williams and Roggenbuck (1989) and Tang (2014) was used to measure the place attachment of residents in the gateway communities of national parks, including place dependence and place identity. Semantic adjustments and question item deletions were made in accordance with the actual situation of the research, and a total of nine question items were included after correction. Environmental commitment was primarily assessed with reference to the scale proposed by Davis et al. (2008), Davis et al. (2011), Su et al. (2019), and Ozbey et al. (2024) which contained a total of 11 items, and “I strongly agree with the content of the community’s slogans, bulletin boards, cultural walls, and radio broadcasts about environmental protection” was added according to the actual situation of the research site. Environmentally responsible behaviors were measured using the same items as used by Vaske and Korin (2001), Halpenny (2010), Chen et al. (2021), and Liu et al. (2022) in their studies, with the addition of “I can refrain from harming the flora and fauna of China National Parks of Hainan Tropical Rainforest.” All scales were measured on a five-point Likert scale (1 = strongly disagree; 5 = strongly agree). The questionnaire has been evaluated by experts and scholars for many times, and has good reliability and validity in the prediction test, indicating that the questionnaire can be used for formal research. We explained to the community residents before filling in the questionnaire that the “environment” in the questionnaire refers to the environment including the community and the national park around the community, so as to guide the residents to think about their attitude towards the environmental protection of the national parks, and avoid the deviation of research results caused by the difference in residents’ definition scope of “environment.”

**Table 2:** Questionnaire items and sources.

Variables	Items		Questionnaire sources
Place dependence (PD)	PD01	I feel that I cannot leave this community and the people in this neighborhood.	Williams and Roggenbuck (1989) and Tang (2014)
	PD02	Living in this community gives me more satisfaction than living in other places.	
	PD03	This community has provided me with living conditions that are not available anywhere else.	
	PD04	I can always get help in this community when I am in trouble.	
Place identify (PI)	PI01	I am proud and honored to live in this community.	
	PI02	Unless I’m out on business, I usually prefer to stay in the community.	
	PI03	When I’m out and about, I often think of this community where I live.	
	PI04	I like this community more than any other place.	
	PI05	I have never thought of moving out of this community to live elsewhere.	
Environmental commitment (EC)	EC01	I am interested in strengthening my connection to the environment in the future.	Davis et al. (2008), Davis et al. (2011), Su et al. (2019), and Ozbey et al. (2024)
	EC02	I feel closely connected to my environment.	
	EC03	When I make plans for myself, I consider the impact my decisions may have on the environment.	
	EC04	In my opinion, humans and the environment are interdependent.	
	EC05	I feel good when things happen that are good for the environment.	
	EC06	Feeling connected to my environment is important to me.	
	EC07	I wish I could always feel a strong connection to my environment.	
	EC08	I am likely to feel a connection to my environment in the future.	
	EC09	I am very attached to the natural environment.	
	EC10	I feel it is my duty to have the best interests of the environment at heart.	
	EC11	I strongly agree with the contents of the community’s slogans, bulletin boards, cultural walls and broadcasts about environmental protection.	
Environmentally responsible behavior (ERB)	ER01	I will try to learn how to solve environmental problems around me	Vaske and Korin (2001), Halpenny (2010), Chen et al. (2021), Liu et al. (2022)
	ER02	I’ll be talking to others about the environmental issues here.	
	ER03	I’ll try to convince my friends to protect the environment here.	
	ER04	I will report to the government if I see someone destroying the environment of my community.	
	ER05	I follow the rules and do not damage the environment.	
	ER06	If there’s an event here to protect the environment, I’ll join it.	
	ER07	I can refrain from harming the flora and fauna of China National Parks of Hainan Tropical Rainforest.	

### 4.3 Data collection

The data in this paper were collected from June to August 2023 via a combination of offline and online questionnaires. The distribution of offline questionnaires mainly took the form of a household survey in which residents were asked to complete the questionnaires, and mainly covered the villages of Maona, Fanglong, Xincun, and others in Shuiman Township, where tourists are more concentrated. While distributing the questionnaires offline, we also contacted the government of Shuiman Township and asked them to distribute the online questionnaires through the WeChat online group, which covered the villages of Yapai and Shuiman in Shuiman Township, where there are fewer tourists. Out of a total of 413 questionnaires distributed in this research, 38 which were not completed correctly or for which the answer time was too short were excluded, and 375 valid questionnaires were obtained, an effective response rate of 90.8%.

The demographic characteristics of the samples collected in this questionnaire survey are shown in Table 3. Of the respondents, 52% were female, 62.93% were aged 25–44, 73.07% were farmers, most (92.53%) had been living there for a long period (more than 10 years), 88.79% had *per capita* monthly household incomes below 2,000 yuan, and 73.87% did not have a high level of education (high school/vocational high school). These characteristics were consistent with the actual situation in the case study site.

**Table 3:** Sample demographic characteristics.

Variables	Groups	Frequency	%	Variables	Groups	Frequency	%
Gender	Female	195	52.00	Length of residence	Less than 1 year	2	0.53
	Male	180	48.00		1–3 years	8	2.13
Age	Below 14	4	1.07		3–10 years	18	4.80
	15–24	75	20.00		More than 10 years	347	92.53
	25–44	236	62.93	Education	Junior high school or below	120	32.00
	45–64	55	14.67		High school/vocational high school	157	41.87
	Over 65	5	1.33		Three-year college	81	21.60
Career	Peasant	274	73.07		Undergraduate or above	17	4.53
	Student	48	12.80	Monthly household incomes	Below RMB1000	95	25.33
	Individual business	19	5.07		RMB 1001–2000	238	63.46
	Worker of an enterprise	16	4.27		RMB 2001–3,000	31	8.27
	Government employee	10	2.67		RMB3001-4000	11	2.93
	Public institution employee	3	0.80				
	Others	5	1.33				

## 5 Results

### 5.1 Tests of normality, reliability, validity, and common method bias

The mean value of each question item in the scale used in this study was more than 3.0, indicating that the residents of the national park gateway community have developed a certain degree of place dependence, place identity, environmental commitment, and environmentally responsible behavior. The normality test found that the skewness of the sample ranged from −0.746 to −0.157, and the kurtosis ranged from −0.959 to 1.011, with absolute values of less than 1.96, indicating that the sample could be accepted as normally distributed at the 95% confidence level.

SPSS 24.0 software was used to test the reliability and validity. As shown in Table 4, the Cronbach’s coefficient of each variable was between 0.810 and 0.909, and the combined reliability was between 0.813 and 0.909, which satisfies the reliability requirement of 0.7 (Nunnally and Bernstein, 1994), indicating that the internal consistency of the questionnaire was good. The factor loadings of each question item ranged from 0.631 to 0.785, satisfying the requirement of greater than 0.5, and the AVE ranged from 0.478 to 0.522, in which the two variables of place dependence and place identity were higher than the standard of 0.5, and the environmental commitment and environmentally responsible behaviors were slightly lower than 0.5, which was acceptable (Zhang and Zheng, 2021). Fornell’s method was used to test the discriminant validity (Fornell and Larcker, 1981). The test results are shown in Table 5. The square root of the AVE of each variable was greater than the correlation coefficient between the variable and other variables. The maximum value of the heterotrait–monotrait ratio was 0.812 which was below the threshold value of 0.85, indicating that the discriminant validity of this questionnaire met the requirements.

**Table 4:** Results of the measurement model analysis.

Variables	Items	Factor loading	CR	AVE	Cronbach’s α
Standard		>0.5	>0.7	>0.5	>0.7
PD	PD01	0.785	0.813	0.522	0.810
	PD02	0.764			
	PD03	0.681			
	PD04	0.663			
PI	PI01	0.72	0.838	0.509	0.837
	PI02	0.727			
	PI03	0.732			
	PI04	0.682			
	PI05	0.709			
EC	EC01	0.719	0.909	0.478	0.909
	EC02	0.668			
	EC03	0.74			
	EC04	0.652			
	EC05	0.652			
	EC06	0.714			
	EC07	0.705			
	EC08	0.736			
	EC09	0.631			
	EC10	0.716			
	EC11	0.662			
ERB	ER01	0.717	0.865	0.479	0.865
	ER02	0.653			
	ER03	0.735			
	ER04	0.702			
	ER05	0.711			
	ER06	0.656			
	ER07	0.669			

**Table 5:** Results of discriminant validity test.

Variables	PD	PI	EC	ERB
PD	0.722			
PI	0.615	0.713		
EC	0.55	0.49	0.691	
ERB	0.584	0.69	0.671	0.692

The diagonal numbers are the square root of the variable AVE.

To minimize common method bias, this study semantically adjusted the questionnaire items for easier comprehension and collected the questionnaires in a multitemporal, multisite, and anonymous manner. Harman’s one-factor method was used to test the results, and this showed that the variance explained by the first factor before rotation was 39.126%, which was below the 40% criterion and indicated that the sample obtained in this paper did not have a significant common method bias.

### 5.2 Evaluation of structural equation modeling and hypothesis testing

#### 5.2.1 Evaluation of structural equation modeling

Amos 24.0 software allowed the model fit effect to be tested and corrected. The final results of the main evaluation indexes of model fit are shown in Table 6: χ^2^/df between 1 and 3 met the requirements; RMSEA was less than 0.1, RMR was less than 0.05; GFI, AGFI, CFI, NFI, and TLI were all greater than 0.9, which indicated that the sample data of this study had a good fit with the proposed model.

**Table 6:** Results of the model fit.

Indicators	χ^2^	df	χ^2^/df	GFI	AGFI	RMSEA	RMR	CFI	NFI	TLI
Threshold	–	–	1 < NC < 3	>0.9	>0.9	<0.10	<0.05	>0.9	>0.9	>0.9
Value	434.947	305	1.426	0.920	0.901	0.034	0.026	0.973	0.917	0.969
Result	Acceptable	Acceptable	Acceptable	Acceptable	Acceptable	Acceptable	Acceptable	Acceptable	Acceptable	Acceptable

#### 5.2.2 Hypothesis testing

The results obtained from hypothesis testing using Amos 24.0 software are shown in Table 7. The results showed that the direct effect of place dependence on environmentally responsible behavior was not significant (*β* = −0.028, *p* > 0.05), and H1a was not supported; place identity could directly, positively, and significantly affect environmentally responsible behavior (*β* = 0.596, *p* < 0.001), supporting H1b; place dependence could positively and significantly affect place identity (*β* = 0.733, *p* < 0.001), supporting H2; both place dependence and place identity had positive and significant effects on environmental commitment (*β* = 0.475, *p* < 0.001; *β* = 0.221, *p* < 0.05), supporting H4a and H4b; and environmental commitment had a significant positive effect on environmentally responsible behavior (*β* = 0.422, *p* < 0.001), supporting H5.

**Table 7:** Results of hypothesis test.

Hypothesis	Unstandardized path coefficients *β*	Standardized path coefficient *β*	S.E.	*t* value	Result
H1a:PD → ERB	−0.024	−0.028	0.063	−0.386	Not supported
H1b:PI → ERB	0.507***	0.596***	0.065	7.841	Supported
H2:PD → PI	0.752***	0.733***	0.065	11.487	Supported
H4a: PD → EC	0.404***	0.475***	0.079	5.139	Supported
H4b: PI → EC	0.183*	0.221*	0.072	2.554	Supported
H5: EC → ERB	0.432***	0.422***	0.06	7.166	Supported

***p < 0.001, *p < 0.05.

#### 5.2.3 Mediating effects test

In this study, we used the bootstrap method in the AMOS 24.0 software to test the mediating effect of place identity and environmental commitment, and the chain mediating effect of “place identity–environmental commitment,” and the test results are shown in Table 8. The total effect of place dependence on environmentally responsible behavior was significant (*β* = 0.591), within which the direct effect was not significant, proving again that H1a was not valid, whereas the total indirect effect was significant (*β* = 0.615). The indirect effect worked through three mediating paths: place dependence influenced environmentally responsible behavior through the mediating of place identity (*β* = 0.381), supporting H3; place dependence influenced environmentally responsible behavior through the mediating of environmental commitment (*β* = 0.175), supporting H6; and place dependence influenced environmentally responsible behavior through the chain mediating of “place identity–environmental commitment” (*β* = 0.06), supporting H7.

**Table 8:** Results of bootstrap mediating effects test.

Path	*β*	SE	Bias-corrected 95% CI		Percentile 95% CI		Result
			Lower	Upper	Lower	Upper	
H3:PD → PI → ERB	0.381	0.057	0.286	0.517	0.281	0.509	Supported
H6: PD → EC → ERB	0.175	0.047	0.103	0.295	0.099	0.281	Supported
H7: PD → PI → EC → ERB	0.06	0.03	0.011	0.131	0.003	0.118	Supported
Total indirect effect	0.615	0.082	0.48	0.819	0.47	0.797	
Direct effect	−0.024	0.082	−0.192	0.138	−0.186	0.144	
Total effect	0.591	0.072	0.46	0.742	0.461	0.743	

## 6 Conclusion and implications

### 6.1 Conclusion and discussions

In this study, we introduced environmental commitment, which had been less discussed in previous studies, as a mediating variable to further explain the influence of place attachment on environmentally responsible behavior, construct a theoretical model, and explore the mechanism for generating environmentally responsible behavior among residents in communities at the gateway of the Hainan Tropical Rainforest China National Park, based on the results of the field research in Shuiman Township, and reached the following four conclusions.

First, the place attachment of gateway community residents positively affects their environmentally responsible behavior. Of these, the direct impact of place dependence on environmentally responsible behavior is not significant, but place dependence indirectly affect behavior via the mediating effect of place identity. This study again verified the influence of place attachment on environmentally responsible behavior and the relationship between place dependence and place identity and clarified the influence mechanism of “place attachment → place identity → environmentally responsible behavior.”

The reason why place dependence could not directly affect environmentally responsible behavior in this study is that, although Shuiman Township is located at the gateway of the National Park, the transportation to and from Wuzhishan City and major cities in Hainan is smooth, and communication facilities are available, so the residents have close communication with the outside world. Although the gateway community residents have mostly lived here for generations and are deeply emotionally connected to the community and the national park, however, due to objective factors such as underdeveloped industries, relatively low incomes, and a lack of cultural life, residents have to leave their hometowns to seek better living conditions in the cities, so they are less dependent on local community. As a result, their functional dependence on the local area is weaker and therefore unable to exert a significant influence on their environmentally responsible behavior. Gateway communities are often located on the periphery of national parks, such as the Giant Panda National Park and the Shennongjia National Park (Cui et al., 2021; Ma et al., 2017), and with convenient transportation and basic communication facilities, the place dependence on gateway community residents may be less strong, and their material needs cannot be satisfied, and therefore environmentally responsible behavior is not effectively stimulated. This finding also underlines the important value of place identity, extending the use of the findings of Qu et al. (2020) to residents of the gateway community.

Place dependence is an external functional demand, which is more inclined to the unilateral material demand for a place on the part of the individual and does not involve emotional interaction, whereas place identity is a heartfelt emotional acceptance of a place, which represents a deeper level of emotional attachment. Their emotional attachment to the local area remains strong because of the influence of the Chinese native culture, blood, geographic ties, etc. (Xiang et al., 2022). It is found that place dependence has an impact on place identity and environmental commitment, indicating that place dependence, the most basic need and attachment of residents to the community, is the root driving force of residents’ psychological and behavioral motivation. It turns out that compared to local dependence, place identity is more likely to encourage residents to take the initiative to implement environmentally responsible behaviors (Liu et al., 2017), demonstrating the strong driving force of place identity on individual behavior.

Second, place attachment positively and significantly affects the environmental commitment of gateway community residents. The connection between place attachment and environmental commitment has received minimal attention from scholars. Tournois and Rollero (2020) confirmed the positive effect of place attachment on commitment from the perspective of long-term residence intention. This study explored the impact of place attachment on environmental commitment from the perspective of environmental protection, which compensates for the omission of the relationship between the two in previous research. Place attachment and environmental commitment are two important psychological factors predicting individual behavior, and the attachment of residents to a place strengthens their ties with it via the spillover effect that generates environmental commitment.

Third, the environmental commitment of gateway community residents positively and significantly influences their environmentally responsible behavior. Environmental commitment, as an affective commitment, has a direct contribution to nondirected voluntary behavior (Cooper and Viswesvaran, 2005). Environmental commitment reflects the dependence of an individual on the environment, their sense of responsibility, and their identification with environmental protection and is an intrinsic motivation for residents to implement environmentally responsible behavior. This study demonstrates that environmental commitment is an antecedent variable of environmentally responsible behavior and that residents with a stronger commitment to protecting the environment are more likely to exhibit environmentally responsible behavior in reality. This study also demonstrates that the relationship between the two is supported by data gathered from residents of the studied national park gateway community, expanding the scope of the application of environmental commitment.

Finally, the environmental commitment of the gateway community residents plays a mediating role in the influence of place dependence on environmentally responsible behavior, and together with place identity, it constitutes a “place identity–environmental commitment” mediating.

Most of the current research on environmentally responsible behavior fails to consider the influence of environmental commitment on environmentally responsible behavior; there are also fewer studies on the antecedent variables of environmental commitment. Although previous scholars have studied some aspects of the research framework presented in this paper to varying degrees, few have tested the structural relationships between these variables simultaneously. This study confirms Lee’s (2011) model of “place attachment → environmental commitment → environmentally responsible behavior” for tourists and extends it to residents of national park gateways, and also provides a more in-depth interpretation of the relationship between the two dimensions of place attachment based on the performance of environmentally responsible behaviors by residents.

This study reveals the mechanism by which place attachment affects environmentally responsible behavior through environmental commitment and the mediating role of “place identity–environmental commitment,” expands the scope of application of environmental commitment, proposes a new path of environmentally responsible behavior, and enriches the research on environmentally responsible behavior. Environmental commitment is widely used in the study of the green behavior of enterprises (Xing et al., 2020). The value of environmental commitment in the environmentally responsible behavior of tourists and residents of tourist destinations merits further exploration by scholars.

### 6.2 Practical insights

National park gateway communities are the first stop for visitors to national parks, and they fulfil not only economic functions such as exhibitions and services but also ecological functions such as environmental protection, acting as the link between the national park, community residents, and visitors. As the owners of national parks, the environmentally responsible behaviors of gateway community residents significantly affect the ecological sustainability and high-quality development of national parks. This study proposes the following three management strategies based on the main factors affecting the environmentally responsible behavior of gateway community residents, including place attachment and environmental commitment.

The first strategy is to deepen the place dependence of residents in the gateway community through industrial revitalization. This study found that place attachment is the basis for environmental commitment and environmentally responsible behavior and is an endogenous motivation for residents to create a sustainable lifestyle. Strengthening the place attachment of community residents begins with deepening place dependence. Industrial revitalization is the foundation of rural revitalization and an important means by which the living needs of residents can be met and their functional dependence on place increased. It is necessary for the gateway communities of national parks to carefully cultivate characteristic industries according to local conditions, make full use of the brand value of national parks, promote the income of community residents through industrial development, and encourage more residents to return to their hometown. For the study area, the community managers can accelerate the development of tea, Li rice wine, Li brocade and other characteristic ecological industries, actively develop rural tourism, and promote the integration of agriculture, culture and tourism.

The second strategy is to promote a strong sense of place identity among residents in the gateway community through cultural cultivation. Cultural identity is the deepest level of identity. To stimulate the place identity of residents, it is necessary to build a local cultural system that is generally recognized by residents. It is important to support and encourage the organization of folk festivals and events, protect and promote local traditional culture, and build a common spiritual home in the community. For our research site, the community managers should support and encourage the holding of long table banquet, Li and Miao New Year, early spring tea picking festival, National Park Day and other special folk festival activities, protect and promote local culture, enhance residents’ cohesion and pride, and promote residents’ comprehensive quality with cultural confidence.

The third strategy is to establish an environmental protection concept, guiding residents to proactively make environmental commitments. This study determined that environmental commitment is a bridge connecting place attachment and environmentally responsible behavior. Through environmental protection publicity, environmental public welfare activities, and other methods close to the lives of the general public, communities enable residents to feel that they and the national park are a closely linked community with strong emotional attachments. Transforming place attachment into environmental commitment also requires mobilizing the power of the general public for ecological governance and protection. Both hard and soft measures should be taken to prevent behaviors that harm the ecological environment, vigorously advocate the construction of eco-friendly communities, teach easy-to-use environmental protection skills, so that community residents will consciously form and abide by their environmental commitments, transforming these verbal environmental commitments into practical environmentally responsible behaviors.

## Data Availability

The raw data supporting the conclusions of this article will be made available by the authors, without undue reservation.
